# Evaluation of a Retrieval-Augmented Generation-Powered Chatbot for Pre-CT Informed Consent: a Prospective Comparative Study

**DOI:** 10.1007/s10278-025-01483-w

**Published:** 2025-03-21

**Authors:** Felix Busch, Lukas Kaibel, Hai Nguyen, Tristan Lemke, Sebastian Ziegelmayer, Markus Graf, Alexander W. Marka, Lukas Endrös, Philipp Prucker, Daniel Spitzl, Markus Mergen, Marcus R. Makowski, Keno K. Bressem, Sebastian Petzoldt, Lisa C. Adams, Tim Landgraf

**Affiliations:** 1 School of Medicine and Health, Department of Diagnostic and Interventional Radiology, Klinikum Rechts Der Isar, TUM University Hospital, Technical University of Munich, Munich, Germany; 2 Institute for Computer Science, Free University of Berlin, Berlin, Germany; 3 School of Medicine and Health, Institute for Cardiovascular Radiology and Nuclear Medicine, German Heart Center Munich, TUM University Hospital, Technical University of Munich, Munich, Germany; 4 Clinic for General, Visceral and Minimally Invasive Surgery, DRK Kliniken Berlin Köpenick, Berlin, Germany

**Keywords:** Artificial intelligence, Medical informatics, Natural language processing, Radiology, Large language models

## Abstract

This study aims to investigate the feasibility, usability, and effectiveness of a Retrieval-Augmented Generation (RAG)-powered Patient Information Assistant (PIA) chatbot for pre-CT information counseling compared to the standard physician consultation and informed consent process. This prospective comparative study included 86 patients scheduled for CT imaging between November and December 2024. Patients were randomly assigned to either the PIA group (*n* = 43), who received pre-CT information via the PIA chat app, or the control group (*n* = 43), with standard doctor-led consultation. Patient satisfaction, information clarity and comprehension, and concerns were assessed using six ten-point Likert-scale questions after information counseling with PIA or the doctor’s consultation. Additionally, consultation duration was measured, and PIA group patients were asked about their preference for pre-CT consultation, while two radiologists rated each PIA chat in five categories. Both groups reported similarly high ratings for information clarity (PIA: 8.64 ± 1.69; control: 8.86 ± 1.28; *p* = 0.82) and overall comprehension (PIA: 8.81 ± 1.40; control: 8.93 ± 1.61; *p* = 0.35). However, the doctor consultation group showed greater effectiveness in alleviating patient concerns (8.30 ± 2.63 versus 6.46 ± 3.29; *p* = 0.003). The PIA group demonstrated significantly shorter subsequent consultation times (median: 120 s [interquartile range (IQR): 100–140] versus 195 s [IQR: 170–220]; *p* = 0.04). Both radiologists rated overall quality, scientific and clinical evidence, clinical usefulness and relevance, consistency, and up-to-dateness of PIA high. The RAG-powered PIA effectively provided pre-CT information while significantly reducing physician consultation time. While both methods achieved comparable patient satisfaction and comprehension, physicians were more effective at addressing worries or concerns regarding the examination.

## Introduction

Large Language Models (LLMs) have emerged as transformative tools in medicine, offering applications ranging from clinical decision support to patient education [1]. Their ability to process and generate human-like language has unlocked new possibilities, including streamlining communication and automating routine clinical tasks, such as anamnesis, structured reporting, text translation, summarization, differential diagnoses, decision support, referrals, and data extraction [2]. One particularly promising application is the use of LLMs to facilitate electronic informed consent (e-consent), where their capacity for clear and personalized communication can address common challenges, such as ensuring patient comprehension of complex medical procedures [3]. However, despite their potential, concerns about the accuracy, context-awareness, and reliability of LLMs limit their application in sensitive domains like informed consent [4].

Recent studies have begun to explore the utility of LLMs in creating and delivering consent materials. For example, one study evaluated Generative Pre-Trained Transformers (GPT)−3.5-generated information on risks, benefits, and alternatives for surgical procedures, demonstrating improved readability and accuracy compared to those generated by surgeons [5]. Another study demonstrated that GPT-4-generated consent materials can improve readability and accessibility while maintaining essential medical and legal content [6]. Furthermore, one investigation assessed GPT-3.5 and GPT-4 in generating patient-specific informed consent documents for nuclear medicine procedures [7]. While GPT-4 showed improvements in accuracy, appropriateness, and detail over GPT-3.5, hallucinations, and insufficient domain specificity were reported.

To address these limitations, Retrieval-Augmented Generation (RAG) has been introduced as a means of anchoring LLM outputs in validated, domain-specific information. RAG operates by retrieving the most relevant content from a structured knowledge base and supplying it to the LLM for response generation, ensuring that the generated output remains accurate and contextually appropriate [8]. This approach has demonstrated particular utility in fields requiring precision and reliability, such as legal documentation and patient education [9, 10].

In radiology, obtaining informed consent is a time-intensive process. For example, a study evaluating consent for intravascular contrast administration found that physician counseling required an average of 11.4 min per patient, accounting for approximately 7% of a radiologist’s professional time in a busy department [11]. Thus, with a daily caseload of approximately 30 patients, this translates to 5.7 h and roughly $1,282.50 in daily labor costs, based on an average radiologist hourly rate of $225 [12].

Enhancing the informed consent process with RAG-powered chat applications offers a viable solution. By providing patients with information about the examination prior to the doctor consultation, these tools can reduce the time required for counseling while mitigating the risks of standalone LLMs generating inaccurate or misleading responses. As a result, this approach has the potential to significantly alleviate the time burden on radiologists by ensuring the delivery of patient-specific and accurate information about the examination prior to the radiologist’s counseling and obtaining informed consent.

Therefore, in this prospective comparative study, we developed a RAG-based Patient Information Assistant (PIA) chat application for pre-CT information counseling and evaluated its feasibility, usability, and effectiveness as a precursor to the standard physician consultation and informed consent process compared to the standard doctor-led consultation prior CT.

## Material and Methods

### Study Design

This monocentric prospective comparative study developed and leveraged a RAG-powered chat application named PIA, evaluating the feasibility, usability, and effectiveness of integrating PIA for obtaining pre-CT informed consent into the clinical workflow between November and December 2024. Participants were randomly assigned to one of two groups. In the PIA group, participants first received pre-CT information via the PIA chat app on an iPad, after which they were asked to answer questions about satisfaction, clarity and comprehension of information, and concerns. Afterward, they underwent the standard physician consultation and informed consent process required for legal reasons. In contrast, the control group received only the standard pre-CT physician consultation and informed consent, which is also routinely done via iPad at our institution, and were then asked the identical questions. If patients could not use the iPad themselves, a staff member assisted in filling out the responses. However, in the intervention group, this assistance did not include any additional explanation of the procedure beyond what was provided by the chatbot. The sample size was determined with a priori power analysis for an independent-sample t-test (two-sided α = 0.05, power = 0.80) to detect a moderate effect size of Cohen’s d = 0.60. This effect size corresponds to a one-point difference on the ten-point Likert scale questions. Based on this calculation, a total of 90 participants (45 per group) were recommended. Ethical approval was granted by the institutional review board of the [anonymized] (2024–469-S-KK). All participants were informed about the study’s purpose and were required to provide written informed consent for their anonymized data to be included in the study prior to enrollment.

### Inclusion and Exclusion Criteria

Eligible participants were individuals aged 18 years or older who were scheduled for CT imaging as part of standard diagnostic or clinical care at the [anonymized] on November 11, November 12, December 9, or December 10, 2024. Individuals with severe cognitive or physical impairments that interfered with their ability to answer questions about baseline characteristics or Likert-scale questions about clarity of information, concerns, and satisfaction, who had participated in the study during a previous CT scan, or who were unwilling or unable to provide informed consent were excluded from the study.

### Variables

Baseline characteristics included age in years, sex (male and female), highest educational level (none, lower secondary school diploma, intermediate secondary school diploma, high school diploma, or university degree), self-reported use of technical devices (never, rarely, more than once a week, several times a week, or daily), and self-reported health status (very poor, poor, sufficient, good, or very good). We developed six ten-point Likert-scale questions to compare the perceived clarity of information, reduction of concerns, level of information, and satisfaction with information in the PIA group after information counseling with PIA alone versus after the doctor-led counseling and informed consent process alone in the control group. The questions addressed participants’ satisfaction with the method used to deliver the information, the extent to which the information helped to reduce concerns or worries, whether participants still had concerns or worries regarding the upcoming examination, how well-informed they felt about their upcoming treatment, the extent to which the provided information covered all necessary aspects of the examination, and the clarity of the information provided regarding the upcoming CT scan. Additionally, we recorded the duration of the doctor consultation and informed consent discussion, following either the use of the PIA or the standard doctor consultation alone. After completing the doctor consultation and informed consent discussion, patients in the PIA group were asked to indicate their preference for pre-CT informed consent: PIA, doctor consultation, or both equally, which was the only question asked after both counseling methods were used. Finally, two radiologists with eight (R1) and nine (R2) years of experience in cross-sectional imaging were asked to rate each PIA chat independently regarding overall quality (poor, fair, good, very good, excellent), scientific and clinical evidence (strongly disagree, disagree, neutral, agree, strongly agree), clinically usefulness and relevance (strongly disagree, disagree, neutral, agree, strongly agree), consistency (yes, undecided, or no), and up-to-dateness (yes, undecided, or no) of responses, as established in a previous study by Truhn et al. [13].

### 1Design of the PIA Chat App

**Fig. 1:**
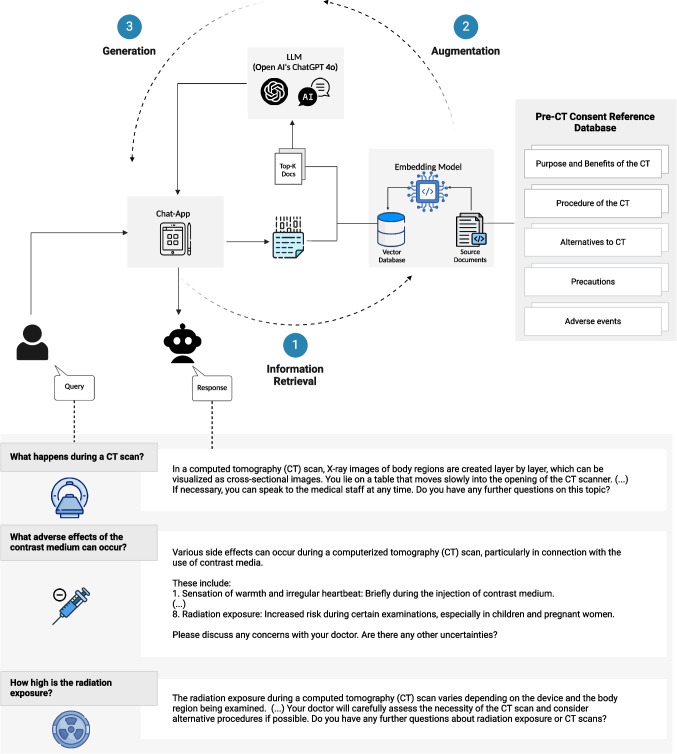
Schematic illustration of the workflow for the Patient Information Assistant (PIA) chat application. After a patient submits a query through the PIA chat app, the query is encoded and compared against a vector database built from the embeddings of documents within the pre-CT consent reference database. This database contains key information categories, including the purpose and benefits of the CT, the procedure of the CT, alternatives to CT, precautions, and potential adverse events. The system retrieves the most relevant documents based on semantic similarity to the query. These retrieved documents, along with the user query, are then provided as input to the large language model (LLM), OpenAI’s Chat Generative Pre-trained Transformers (GPT) 4o. This step augments the LLM’s contextual understanding, allowing it to generate an accurate and contextually appropriate response. Finally, the LLM produces a user-friendly response, which is delivered back to the chat app and displayed to the user. This integrated process leverages retrieval-augmented generation to combine structured information retrieval with advanced language generation, ensuring accurate and personalized patient support. As a reference for the reader, we present examples of actual conversations between patients and the PIA chat app during pre-CT informed consent translated into English below

Figure 1 shows a schematic representation of the workflow for the PIA chatbot. PIA is a RAG-powered chatbot designed to facilitate pre-CT informed consent in German. It integrates OpenAI’s ChatGPT 4o with a structured retrieval mechanism to provide accurate, context-specific answers to patient queries [14]. The knowledge base, derived from pre-CT information sheets and databases, is divided into discrete paragraphs, and semantic embeddings are generated for each using the German-language model mxbai-embed-large-v1 from Mixedbread, deployed on local servers to ensure data privacy [15]. These embeddings enable the system to identify the most relevant content for each patient query through similarity comparisons. When a patient submits a query via the PIA chat app, the system encodes the query into an embedding and compares it with the pre-CT knowledge base. The most semantically similar paragraphs are retrieved and supplied to ChatGPT 4o, which is accessed through an application programming interface. Using this context, the LLM generates a response grounded in the retrieved information, ensuring both accuracy and relevance. The response is delivered through a chat interface, giving patients the option to read it themselves or use the text-to-speech feature to have PIA read the response aloud. The PIA system focuses on key information categories, including the purpose and benefits of the CT, procedural details, alternatives, precautions, and potential adverse events. This architecture ensures that responses are not only precise but also tailored to the specific informational needs of patients. The integration of RAG allows PIA to enhance patient understanding while maintaining reliability, clinical relevance, and correctness. Real-world examples of conversations captured during the study and translated into English are shown in Fig. 1.

### Statistical Analysis

All statistical analyses were performed using Python 3.10.12, employing the libraries Matplotlib (3.8.0), Numpy (1.26.4), Pandas (2.2.2), Scipy (1.13.1), and Statsmodels (0.14.4) [16–20]. Normal distribution was assessed using the Shapiro–Wilk test, and as all variables were non-normally distributed, numerical data were summarized as medians with interquartile ranges (IQR), except for the 10-point Likert-scale questions, which were reported as means with standard deviations (SD) to better illustrate differences between the PIA and control group. Categorical variables were reported using absolute counts and percentages. Response rates were calculated by documenting all instances where patients were invited to participate in the study and determining the percentage of those who agreed to participate. Comparison of baseline characteristics between the PIA and control group was performed using the chi-squared or Mann–Whitney U test. Differences in responses to Likert-scale questions and the duration of pre-CT consultations were analyzed using the Mann–Whitney U test. In the PIA group, associations between baseline characteristics and patient preferences for PIA, doctor consultation, or both were examined using multinomial logistic regression, with odds ratios (ORs) calculated and equal preference as the reference category. Missing responses on baseline characteristics led to the exclusion from the regression analysis. Statistical significance was determined using a two-sided *p*-value threshold of < 0.05.

## Results

### Study Cohort

**Fig. 2:**
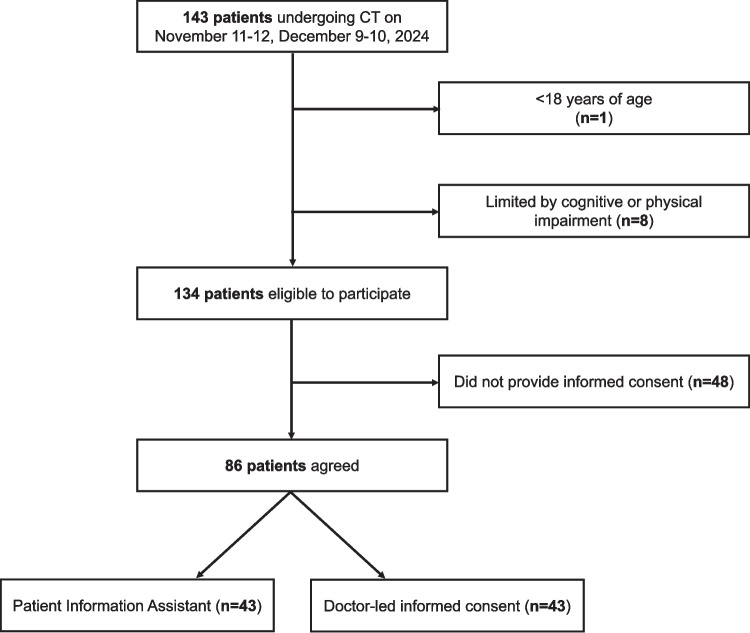
Study enrollment flowchart illustrating the inclusion and exclusion of participants

Of 143 patients who underwent CT scans between November and December 2024, a total of 134 eligible patients were invited to participate in the study, of whom 86 agreed, resulting in a response rate of 64.18%, see Fig. 2. Participants were evenly divided between the PIA group (*n* = 43) and the control group (*n* = 43). The median age of the overall cohort was 60 years (IQR: 49–69), with a median age of 60 years (IQR: 50–69.5) in the PIA group and a median age of 61 years (IQR: 49.5–69) in the control group. Patients were predominantly male (58.14%, *n* = 50), comprising 60.47% (*n* = 26) of the PIA group and 55.81% (*n* = 24) of the control group. Females represented 41.86% (*n* = 36) of the total population, distributed as 39.53% (*n* = 17) in the PIA group and 44.19% (*n* = 19) in the control group.

Among the 77 participants who reported their highest educational level, 36.56% (*n* = 28) held a university degree, 28.57% (*n* = 22) had an intermediate secondary school diploma, 16.88% (*n* = 13) each reported holding a high school diploma or lower secondary school diploma, and 1.3% (*n* = 1) reported no formal education.

Regarding the use of technical devices, 87.06% (*n* = 74) of the 85 respondents reported daily use, 5.88% (*n* = 5) used such devices several times a week, and 3.53% (*n* = 3) used them more than once a week. Another 3.53% (*n* = 3) indicated they never used technical devices, while none reported rare usage.

In terms of self-reported health status, very poor health was reported by 18.82% (*n* = 16) of participants, poor health by 24.71% (*n* = 21), sufficient health by 25.88% (*n* = 22), good health by 18.82% (*n* = 16), and very good health by 11.76% (*n* = 10). There were no significant differences in baseline characteristics between both groups; see p-values and an overview of baseline characteristics for the entire cohort and stratified by PIA and control group in Table 1.

**Table 1:** Overview of baseline characteristics for the total cohort and for the Patient Information Assistant (PIA) and doctor-consultation-only control groups, respectively

Variable	Total	PIA	Control group	*p*-value comparison
Age (*N* = 86)				0.680
Median years (IQR)	60.0 (49.0–69.0)	60.0 (50.0–69.5)	61.0 (49.5–69.0)	
Sex (*N* = 86)				0.660
Male, *n* (%)	50 (58.14%)	26 (60.47%)	24 (55.81%)	
Female, *n* (%)	36 (41.86%)	17 (39.53%)	19 (44.19%)	
Highest educational level (*N* = 77)				0.197
None, *n* (%)	1 (1.30%)	0	1 (2.56%)	
Lower Secondary School Diploma, *n* (%)	13 (16.88%)	4 (10.57%)	9 (23.08%)	
Intermediate Secondary School Diploma, *n* (%)	22 (28.57%)	15 (39.47%)	7 (17.95%)	
High School Diploma, *n* (%)	13 (16.88%)	6 (15.79%)	7 (17.95%)	
University Degree, *n* (%)	28 (36.56%)	13 (34.21%)	15 (38.46%)	
Use of technical devices (e.g., smartphone or laptop) (*N* = 85)				0.157
Never, *n* (%)	3 (3.53%)	0	3 (6.98%)	
Rarely, *n* (%)	0	0	0	
More than once a week, *n* (%)	3 (3.53%)	2 (4.76%)	1 (2.33%)	
Several times a week, *n* (%)	5 (5.88%)	4 (9.52%)	1 (2.33%)	
Daily, *n* (%)	74 (87.06%)	36 (85.71%)	38 (88.37%)	
Health status (*N* = 85)				0.108
Very poor, *n* (%)	16 (18.82%)	8 (19.05%)	8 (18.60%)	
Poor, *n* (%)	21 (24.71%)	11 (26.19%)	10 (23.26%)	
Sufficient, *n* (%)	22 (25.88%)	12 (28.57%)	10 (23.26%)	
Good, *n* (%)	16 (18.82%)	10 (23.81%)	6 (13.95%)	
Very good, *n* (%)	10 (11.76%)	1 (2.38%)	9 (20.93%)	

*IQR* Interquartile range

### Comparison of Information Clarity, Comprehension, and Impact on Patient Concerns

Both groups rated the clarity of the provided information highly, with mean scores of 8.64 (SD: 1.69) in the PIA group and 8.86 (SD: 1.28) in the doctor consultation group (*p* = 0.82). Similarly, the extent to which the information covered all necessary aspects of the CT examination was rated as 8.67 (SD: 1.83) for the PIA group and 8.81 (SD: 1.87) for the control group (*p* = 0.58), indicating that both methods were equally comprehensive. Patients’ overall feeling of being informed was also comparable, with the PIA group scoring 8.81 (SD: 1.40) and the control group scoring 8.93 (SD: 1.61; *p* = 0.35). Furthermore, satisfaction with the method of delivering information was high in both groups, with mean scores of 8.88 (SD: 1.43) for PIA versus 9.16 (SD: 1.43) for the control group (*p* = 0.14).

**Fig. 3:**
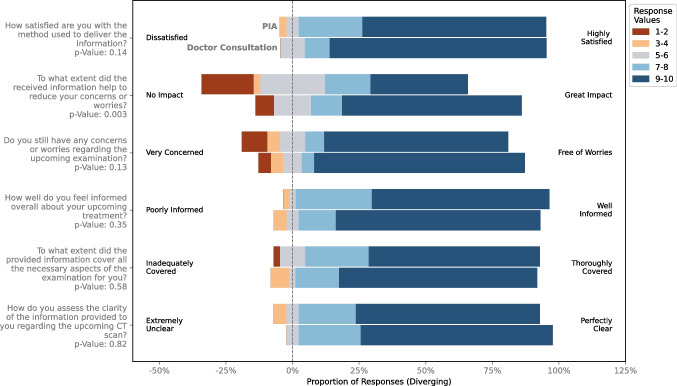
Gantt charts depicting patient responses to ten-point Likert-scale questions following pre-CT informed consent, comparing the Patient Information Assistant (PIA) group with the control group that received doctor consultation for pre-CT informed consent only

While no significant differences were found in how well the information addressed patient concerns or worries (PIA: 8.14 (SD: 2.81) compared to 8.77 (SD: 2.43) in the control group; *p* = 0.13), patients when asked if they still had concerns or worries about the upcoming examination, the doctor consultation group reported slightly fewer concerns, with a mean score of 8.30 (SD: 2.63) compared to 6.46 (SD: 3.29) in the PIA group, which was statistically significant (*p* = 0.003), suggesting that doctor consultations were more effective in alleviating worries. Figure 3 illustrates Gantt charts comparing the results for doctor-only consultations and the PIA group.

### Duration of Pre-CT Consultation

Patients who received PIA prior to the standard pre-CT doctor consultation and informed consent demonstrated significantly shorter consultation times, with a median duration of 120 s (IQR: 100–140) compared to 195 s (IQR: 170–220) for those who underwent the doctor consultation alone (*p* = 0.04).

### Preference for PIA or Doctor Consultation

In the PIA group, 69.77% (*n* = 30) of patients reported equal preference for PIA and the doctor consultation, 11.63% (*n* = 5) preferred PIA over the doctor consultation, and 18.60% (*n* = 8) preferred the doctor consultation over PIA. Multinomial logistic regression analysis revealed no significant associations between baseline characteristics and patient preferences for PIA or doctor consultation compared to equal preference (see Table 2).

**Table 2:** Results of the multinomial logistic regression analysis displaying baseline characteristics and their association with preferences for the Patient Information Assistant (PIA) or doctor consultation within the PIA group

Characteristic	Prefer PIA (*N* = 5)		Prefer doctor consultation (*N* = 8)	
	Odds ratio (95% CI)	*p*-value	Odds ratio (95% CI)	*p*-value
Sex, male	0.47 (0.07–3.26)	0.445	2.12 (0.36–12.34)	0.404
Sex, female	2.13 (0.31–14.73)	0.445	0.47 (0.08–2.75)	0.404
Age	1.02 (0.96–1.09)	0.503	1.02 (0.97–1.07)	0.531
Highest educational level	0.96 (0.50–1.86)	0.913	0.87 (0.50–1.52)	0.632
Use of technical devices	0.83 (0.08–9.13)	0.881	1.46 (0.15–14.64)	0.748
Health status	0.68 (0.28–1.68)	0.402	1.20 (0.58–2.46)	0.619

Equal preference for PIA and doctor consultation for pre-CT informed consent (*N* = 30) served as the reference category. Abbreviation: *CI* Confidence interval

### Radiologist Rating of PIA

**Fig. 4:**
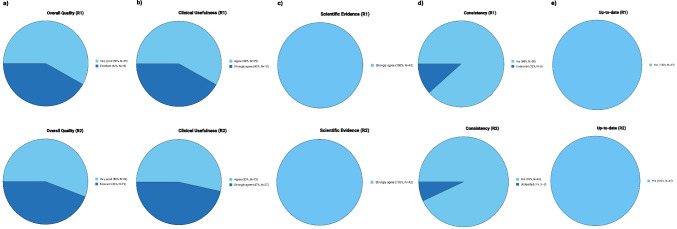
Comparison of Patient Information Assistant (PIA) chat ratings for overall quality (**a**), clinical usefulness (**b**), scientific evidence (**c**), consistency (**d**), and up-to-dateness (**e**) by two radiologists with eight (R1) and nine (R2) years of experience in cross-sectional imaging

For overall quality, the majority of ratings were very good (R1/R2: 58.14% (*n* = 25)/55.81% (*n* = 24)), followed by excellent (R1/R2: 41.86% (*n* = 18)/44.19% (*n* = 19)). Similarly, PIA rated high in clinical usefulness (agree R1/R2: 41.86% (*n* = 18)/46.51% (*n* = 20) and strongly agree R1/R2: 53.49% (*n* = 23)/58.14% (*n* = 25)), while both raters rated all PIA chats within the category scientific evidence with strongly agree (R1/R2: 100% (*n* = 43), respectively). Furthermore, both radiologists rated all responses as up-to-date (100.00%, *n* = 43, respectively). Consistency was also rated highly, with R1 marking 88.37% (*n* = 38) and R2 marking 93.02% (*n* = 40) as “yes,” while a small proportion of chats were rated as “undecided” by R1 (11.63%, *n* = 5) and R2 (6.98%, *n* = 3). Figure 4 illustrates the distribution of ratings across the reviewers.

## Discussion

This study highlights the feasibility and effectiveness of integrating a RAG-powered PIA into the clinical workflow for pre-CT information counseling. Both the PIA and standard doctor consultation groups rated the clarity and comprehension of the information similarly, achieving high scores across all measures of patient understanding and satisfaction. However, only the doctor consultation demonstrated statistically significantly greater effectiveness in alleviating patient worries or concerns compared to the PIA. Conversely, the use of the PIA significantly reduced the duration of the subsequent doctor pre-CT counseling compared to the doctor consultation and informed consent process alone. Furthermore, patient preferences for PIA or doctor consultation showed no association with age, sex, educational level, tech-savviness, or health status, with most patients in the PIA group preferring both methods equally, while radiologists rated the PIA responses high in overall quality, scientific and clinical evidence, clinically usefulness and relevance, consistency, and up-to-dateness. These findings underscore the potential of PIA as a complementary tool to enhance efficiency in the informed consent process while preserving patient satisfaction and comprehension.

Our study’s observation that PIA and physician consultations were rated similarly in terms of clarity and comprehensibility is consistent with the findings of previous studies in other medical domains, where LLMs have even been shown to produce more accessible information. In particular, many consent forms are written at a reading level well above that of the average patient, limiting comprehension and truly informed decision-making [6]. In a case study by Mirza et al., a universal surgical consent form at the largest health care system in Rhode Island that had a Flesch-Kincaid reading level of 12.6th-grade was successfully reduced to a 6.7th-grade reading level using GPT-4, without the introduction of errors or hallucinations [6]. Decker et al. compared GPT-3.5-generated informed consent documents to those authored by surgeons for six common procedures [5]. They found that the mean readability for GPT-generated consent had a reading level of 12.9th-grade compared to 15.7th-grade for surgeon-generated forms, indicating a trend toward improved accessibility. Notably, GPT-3.5 outperformed surgeons in overall completeness and accuracy.

However, one problem with LLMs and their application in medicine that many other studies note is the generation of hallucinated, generic, inconsistent, outdated, or incomplete outputs [21–26] and the lack of providing reliable sources [27]. Therefore, especially in sensitive domains like healthcare, RAG can mitigate such issues and improve the accuracy of the generated information, alleviating problems such as hallucinations and outdated knowledge. For example, a study by Xiong et al. benchmarked RAG systems, finding that grounding LLMs in external knowledge improved medical QA accuracy by up to 18% over traditional chain-of-thought prompting, bringing GPT-3.5 and Mixtral models to near GPT-4 levels of accuracy [28]. Similarly, Ke et al. developed a RAG-powered preoperative counseling model that achieved 91.4% accuracy, outperforming standard GPT-4 responses (80.1%) and approaching human accuracy (86.3%) [29]. Similarly, our paper shows that a RAG-powered PIA adequately combines LLMs with external knowledge sources to generate contextually relevant and accurate responses in the radiological domain.

These findings underscore that retrieval-based AI models may be a viable solution for improving factual consistency in informed consent workflows.

Nevertheless, we found that the PIA was less effective in alleviating patient concerns compared to the standard doctor counseling process. This contrasts with previous literature suggesting that LLMs can generate more empathetic responses than doctors. For instance, a study by Luo et al. evaluating LLM responses to real-world physician–patient messages demonstrated that LLMs can exhibit greater empathy in communication compared to average physician responses [30]. Similarly, Ayers et al. reported that LLMs outperformed physicians in providing higher-quality and more empathetic responses to patient questions in an online forum, highlighting the potential of LLMs to reduce clinician workload and improve patient communication [31].

In contrast to these studies, our work compared real-world physician counseling with a RAG-powered chat application rather than chat-based LLM interactions with patients in an online setting. Physician–patient interactions encompass not only verbal communication but also non-verbal cues, adaptability, and emotional resonance, elements that current LLMs may not replicate effectively [4]. For example, a physician can convey empathy and reassurance through eye contact, body language, and tone of voice, signals that are absent in chatbot interaction. Montague et al. demonstrated that doctors who maintain eye contact are perceived as significantly more likable and empathetic by patients​ [32]. Furthermore, they concluded that the amount of eye contact was the single most important factor influencing patients’ perception of a doctor’s empathy, with adequate eye contact correlating with greater patient trust and follow-through with care​. Such findings underscore that non-verbal social communication is an essential component of the doctor-patient relationship​. By offering attentive presence and appropriate facial expressions or gestures, a physician may alleviate anxiety in ways a chatbot cannot, simply because the machine lacks a human presence. Second, physicians demonstrate adaptability and personalized communication in real-time. During a consent discussion, a human doctor can gauge the patient’s understanding (or confusion) and emotional state and then immediately tailor the conversation accordingly. If a patient looks puzzled or voices a concern, the physician can slow down, rephrase information, or provide additional clarification on the spot. This dynamic feedback loop ensures that patient concerns are addressed as they arise, thereby reducing uncertainty and fear [31]. In contrast, a chatbot typically follows a predetermined script or decision tree; it may not recognize subtle indications of distress or may provide generic responses that do not fully resolve the patient’s specific worries. The flexibility of a live conversation allows physicians to use techniques like the “teach-back” method, where patients are encouraged to repeat information in their own words to confirm understanding [33]. Employing such techniques, clinicians can identify misunderstandings and correct them immediately​ [34]. Notably, the teach-back approach has been shown to improve patients’ knowledge retention and self-care abilities, and its use is associated with better health outcomes across diverse settings​ [34]. This illustrates how interactive, adaptive communication from a physician can ensure the patient truly comprehends the consent information, which in turn builds reassurance. By calibrating the discussion to each patient’s needs, for instance, simplifying complex terms for a layperson or delving deeper into areas the patient is curious about, physicians personalize the consent process. Third, emotional resonance and empathy are more readily conveyed in physician-led interactions, enhancing patient reassurance. A human doctor can listen to a patient’s fears or hesitations and respond with genuine empathy through both words (e.g., “I understand how you feel; many patients find this part worrisome, but…”) and compassionate demeanor. This ability to validate and address emotions helps patients feel understood and supported. The literature strongly supports the impact of provider empathy on patient outcomes: greater physician empathy is correlated with higher patient satisfaction and lower anxiety levels​ [35]. Empathic communication not only comforts the patient in the moment but also builds a foundation of trust in the physician–patient relationship​ [36]. Patients who perceive their doctor as caring and understanding are more likely to believe that their concerns are being taken seriously, which alleviates worry. In the context of informed consent, this means a patient is more apt to voice any last-minute fears to an empathetic physician and be reassured by the response. Empathy also has downstream effects; when patients feel emotionally supported, they tend to have greater confidence in the treatment plan and better adherence to medical advice​ [32, 36]. As a consequence, in our study, the emotional rapport established by physician counselors may have made patients feel safer and more at ease with the procedure, thereby reducing their concerns. This may explain why the PIA—despite its accuracy in providing pre-CT information—was unable to match the ability of physicians to address patient concerns. However, the linguistic style of the chatbot’s response can be controlled by the prompt used by the LLM. In this study, we used a prompt that resulted in short and concise responses to avoid overburdening the patient with information. Therefore, future studies may investigate how different prompts and different response styles may affect patient concerns. The context dependence of LLM performance also highlights the importance of design and application specificity. Models such as WundtGPT, fine-tuned for mental health, leverage psychological principles to enhance empathetic and effective communication [37]. These specialized designs may achieve better results in contexts where emotional support is the primary goal. However, even with such fine-tuning, LLMs remain limited in their ability to dynamically adapt to a patient’s immediate emotional state and nuanced needs, which are critical in high-stakes clinical interactions such as pre-CT counseling [38, 39].

Despite these limitations, our findings suggest that LLMs, particularly when augmented with RAG frameworks, hold substantial potential as complementary tools in the informed consent process. As in our study, a hybrid model in which patients receive preliminary information via a RAG-powered chat app before their physician consultation could streamline workflows, reduce time burdens, and enhance patient comprehension. Moreover, the role of LLMs in augmenting physician-led consultations extends beyond efficiency. Providing patients with accurate, accessible information prior to their consultation empowers them to engage more actively in discussions, ask informed questions, and better understand the procedure [40, 41]. In addition, healthcare providers have full control over the information they provide to the RAG system, creating the opportunity to tailor information to specific local conditions. Unlike generic information that patients may find on the Internet, the information provided by the clinic can be considered trustworthy.

Our study has limitations. First, this was a single-center study conducted within a limited timeframe, which may impact the generalizability of our findings. Second, the study relied on self-reported measures of patient satisfaction, comprehension, and preferences rather than objective assessments, which may introduce bias. Patients may have rated their experiences favorably due to novelty bias or social desirability rather than reflecting actual comprehension or satisfaction. Future studies may incorporate objective assessments, such as knowledge retention or comprehension tests, to further validate our findings. Additionally, the use of a ten-point Likert scale for measuring patient perceptions may not fully capture the complexity of patient experiences. Third, the small sample size may limit the robustness of statistical associations between baseline characteristics and patient preferences for PIA or doctor consultations. Despite our efforts to recruit a larger number of patients, our recruitment period yielded a 64.18% response rate (86 of 134 invited), resulting in a final sample size of 43 participants per group, an estimate that retains approximately 79% power. However, our results demonstrated a statistically significant difference in residual concerns about the upcoming CT examination and consultation times, consistent with our power estimates. Furthermore, no other Likert-scale variable showed an average difference greater than one point between groups, indicating that meaningful comparisons were still feasible with this sample. Future studies may validate our findings in larger samples. In addition, the technology itself poses potential challenges. While RAG mitigates the risks of hallucinations and domain-specific inaccuracies in LLMs, errors in retrieval or model outputs could still lead to incomplete or misleading information. Furthermore, the system’s performance depends heavily on the quality and comprehensiveness of the underlying knowledge base, which requires ongoing maintenance and updates to remain clinically relevant and accurate. However, a key advantage of RAG-based models is that they allow updates to the knowledge base without requiring full model retraining. Potential maintenance strategies can include regular expert-curated updates, version control mechanisms, and human validation [42, 43]. Furthermore, to integrate these updates into routine practice with minimal disruption, best practices include deploying incremental content revisions, scheduling updates during non-peak hours, and maintaining fallback mechanisms for rapid rollback if needed [44, 45]. These strategies may be particularly important for RAG systems that retrieve information for procedures that, unlike CT scans, change periodically, such as written informed consent before chemotherapy when new treatment options are introduced. Notably, RAG systems are more prone to errors when the underlying knowledge base is extensive, as larger datasets increase the complexity of retrieval and synthesis [46]. While our study focused solely on information about CT scans, more complex procedures may require significantly larger and more diverse informational resources and demand even more sophisticated RAG systems to achieve a comparable level of accuracy and completeness, which may be subject to future research.

Finally, regulatory requirements can hinder the integration of such models into real-world clinical practice. At our center, radiologists are required by law to obtain written informed consent from patients before each CT scan, so tools like PIA could only be used as an adjunct to standard physician-led consent.

## Conclusion

In conclusion, we present, to our knowledge, the first RAG-powered PIA for pre-CT information counseling. Our findings demonstrate that PIA effectively informs patients about the CT procedure to a degree comparable to physicians while significantly reducing the time required for the subsequent doctor consultation and informed consent process. Importantly, this approach could be readily adapted to other radiological domains, such as MRI information counseling. However, as PIA was rated less effective than doctor consultations in alleviating patient concerns or worries, our results underscore the irreplaceable value of human interaction in addressing emotional and nuanced patient needs. This suggests that chatbots like PIA should serve to augment rather than replace the role of the physician. To foster chatbot implementation into clinical practice, future efforts should focus on refining chatbot prompts for more empathetic language, exploring adaptive conversation techniques to address patient concerns in real-time, and incorporating emerging multimodal approaches that emulate non-verbal cues. Such advancements may help RAG-powered PIAs more closely replicate the interpersonal elements of physician consultations, further enhancing patient reassurance and satisfaction. Ultimately, future studies may validate the performance of RAG-based health chatbots in larger, more diverse patient populations and integrate robust feedback loops for continuous maintenance and improvement.

## Data Availability

Datasets generated during the current study are available from the corresponding author on reasonable request.
